# Levels and Trends of Esophageal and Stomach Cancer Mortality in Sub-Saharan Africa and the Caribbean

**DOI:** 10.1200/JGO.17.00204

**Published:** 2018-03-05

**Authors:** Temitayo Ogundipe, Mustafa Mustafa, Richard Gillum

**Affiliations:** **Temitayo Ogundipe** and **Mustafa Mustafa**, Howard University Hospital; and **Richard Gillum**, Howard University College of Medicine, Washington, DC

## TO THE EDITOR:

Recent reports of cancer registry data document an extremely high incidence of esophageal cancer but relatively low rates of stomach cancer in sub-Saharan Africa, with favorable trends in the latter.^1,2^ For example, in Malawi, with a 9% HIV prevalence, esophageal cancer (histologically verified in 24%, mainly squamous cell) was the second most common cancer in men and the third most common in women, with a peak rate at 65 years and older, similar age-specific rates in men and women, and higher age-adjusted rates than in other African registries and blacks in the United States.^1^ Because of a lack in comprehensive death registration, death rates were not available. Increases in the incidence of stomach cancer in recent birth cohorts between 1978 and 2007 were seen in Uganda.^2^ We examined mortality estimates from the Global Burden of Disease, Injuries, and Risk Factors Study 2015 (GBD2015) for the causes of these cancers in sub-Saharan Africa and the Caribbean.

The GBD2015 quantified health loss from hundreds of diseases, injuries, and risk factors, using all available data together with multilevel statistical modeling so that health systems could be monitored and disparities quantified. Methods used in the GBD2015 have been described in detail elsewhere.^3,4^

For both sexes and all ages in 2015, the death rates per 100,000 from esophageal cancers were relatively low in the sub-Saharan countries and the Caribbean, with the exception of southern African countries and some Caribbean countries such as Cuba, which had moderate rates. In comparison, China, Mongolia, the United Kingdom, and Greenland had the highest rates (Table 1). For both sexes and all ages, between 1990 and 2015 in sub-Saharan Africa, esophageal cancer death rates showed a slight decrease and then an increase in the eastern and central regions. A slight increase and then a decrease were shown in the southern region.

**Table 1:**
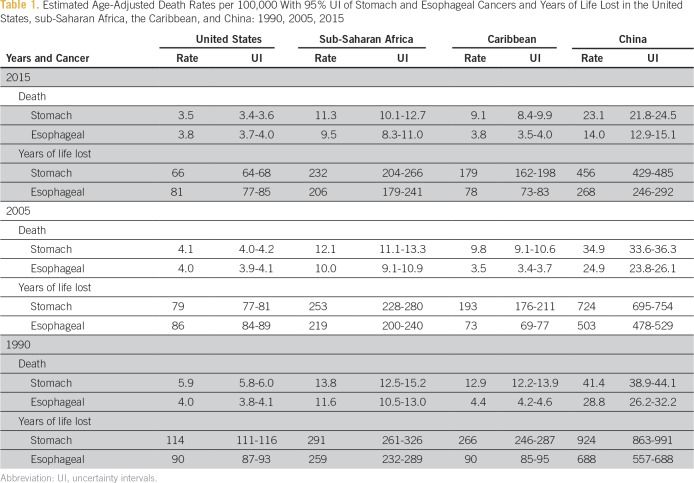
Estimated Age-Adjusted Death Rates per 100,000 With 95% UI of Stomach and Esophageal Cancers and Years of Life Lost in the United States, sub-Saharan Africa, the Caribbean, and China: 1990, 2005, 2015

For both sexes and all ages in 2015, the death rates per 100,000 from stomach cancers were relatively low in the sub-Saharan countries and the Caribbean. In comparison, Japan, China, and Russia had the highest rates in 2015 (Table 1). For both sexes and all ages between 1990 and 2015, stomach cancer death rates showed a slight decline in all regions of sub-Saharan Africa and the Caribbean.

Among men, esophageal or stomach cancer ranked first according to the number of deaths in some African countries.^3^ Stomach cancer ranked in the top five for both sexes in all sub-Saharan African regions, with the exception of the southern region, where only colorectal cancer ranked in the top five.

In conclusion, estimates from the GBD2015 complement data from individual cancer registries, providing useful data for epidemiologic hypothesis generation and public health planning. Future research should explore covariation of the incidence and mortality of esophageal cancer and HIV prevalence and mortality.

## References

[1] Chasimpha SJD, Parkin DM, Masamba L (2017). Three-year cancer incidence in Blantyre, Malawi (2008-2010). Int J Cancer.

[2] Luo G, Zhang Y, Guo P (2017). Global patterns and trends in stomach cancer incidence: Age, period and birth cohort analysis. Int J Cancer.

[3] Global Burden of Disease Cancer Collaboration (2017). Global, regional, and national cancer incidence, mortality, years of life lost, years lived with disability, and disability-adjusted life-years for 32 cancer groups, 1990 to 2015: A systematic analysis for the Global Burden of Disease Study. JAMA Oncol.

[4] GBD 2015 Mortality and Causes of Death Collaborators: Global, regional, and national life expectancy, all-cause mortality, and cause-specific mortality for 249 causes of death, 1980-2015: A systematic analysis for the Global Burden of Disease Study 2015. Lancet 388:1459-1544, 2016 10.1016/S0140-6736(16)31012-1 PMC5388903 27733281

